# Visitor effects on the welfare of captive Sumatran orangutans (*Pongo abelii*) during the pandemic lockdowns

**DOI:** 10.1017/awf.2025.9

**Published:** 2025-03-04

**Authors:** Ezekiel F Gading, Valerie AM Schoof, Maria Franke, Suzanne E MacDonald

**Affiliations:** 1 Department of Psychology, York University, Toronto, Ontario, Canada; 2 Bilingual Biology Program, Multidisciplinary Studies Department, Glendon College, York University, Toronto, Ontario, Canada; 3 Department of Biology, York University, Toronto, Ontario, Canada; 4 Wildlife Conservation & Welfare Science Department, Toronto Zoo, Toronto, Ontario, Canada

**Keywords:** animal welfare, behaviour, COVID-19, human-animal interaction, primates, stress

## Abstract

The COVID-19 pandemic led to unprecedented lockdowns with rippling impacts on the lives of humans and animals alike. Since zoos were among the first institutions to close during the pandemic, the lockdowns presented the opportunity to conduct a natural experiment examining the relationship between visitor presence and the welfare of zoo-housed animals. In this study, we assessed the welfare of six Sumatran orangutans (*Pongo abelii*) at Toronto Zoo both during and following the pandemic lockdowns. We compared behavioural and physiological indicators of welfare during a lockdown and after visitors were reintroduced. Specifically, if the orangutans’ welfare was affected by the visitor re-introduction phase we predicted there would be an increase in the following measures: (1) use of exhibit areas away from visitors; (2) behavioural measures (hiding, self-directed behaviours, agonistic behaviours, agitated movement, and idiosyncratic object-directed behaviours [head slamming, and fabric tearing]); and (3) physiological measures (faecal consistency and glucocorticoid metabolites) when compared to the lockdown. We also measured changes in activity levels such as foraging and inactivity. We found that orangutan exhibit space use did not change when visitors were reintroduced. In fact, the orangutans hid less when visitors were introduced than during the lockdown. Foraging, inactivity, and other behavioural indicators of stress did not change when visitors were introduced. Similarly, neither faecal consistency nor glucocorticoid metabolites changed across the study phases. Our data show that visitor re-introduction did not negatively affect the welfare of the Toronto Zoo orangutans. However, the presence of keepers was found to affect the behaviour of the orangutans and warrants further study.

## Introduction

Prior to the COVID-19 pandemic, it was difficult to imagine a zoo without visitors for weeks let alone months. Zoos primarily fund their daily operations through visitors; thus, visitors are integral to the lives of zoo-housed animals. Hence, should there be negative implications for the welfare of zoo animals, it represents an ethical conflict of welfare with core parts of zoo operations: conservation science and education using live animals (Hutchins *et al.* 2003; Whitworth 2012; Carr 2016; MacDonald & Ritvo 2016a; Patrick & Caplow 2018). It follows that there is a long-standing tradition of zoo-based research concerned with the welfare effects of visitors on the animals in their care, particularly primates (for reviews, see Davey 2007; Sherwen & Hemsworth 2019). Interestingly, there is no single ‘visitor effect’ since visitors exert influence on animal welfare in diverse ways such as noise, visual aspects, size, and mere presence. The effects of visitor presence has been studied in species such as western lowland gorillas (*Gorilla gorilla gorilla*: Wells 2005), lion-tailed macaques (*Macaca silenus*: Mallapur *et al.* 2005), chimpanzees (*Pan troglodytes*: Wood 1998), polar bears (*Ursus maritimus*: Kelly *et al.* 2015), in jaguars (*Panthera onca:* Sellinger & Ha 2005), little penguins (*Eudyptula minor*: Sherwen *et al.* 2015c), koalas (*Phascolarctos cinereus*), and kangaroos (Kangaroo island kangaroos [*Macropus fuliginosus fuliginosus*] & red-necked wallabies, [*Macropus rufogriseus*]: Sherwen *et al.* 2015b). These studies aimed to capture the relationship between stress levels and visitor numbers or presence. For example, in western lowland gorillas, high visitor density was associated with an increase in agonistic behaviours, autogrooming, and abnormal behaviours, all of which were taken as indicators of stress (Wells 2005). While increased visitor counts have been associated with negative changes in behaviour and hormones (e.g. gorillas: Wells 2005), lion-tailed macaques (Mallapur *et al.* 2005), chimpanzees (Wood 1998), the effect is not the same in other species. Some species showed no changes in stress indicators but a degree of ambivalent increase of visual attention towards visitors (Kangaroo island kangaroos & red-necked wallabies: Sherwen *et al.* 2015b), others followed similar negative changes in welfare as in primates (jaguars: Sellinger & Ha 2005); little penguins (Sherwen *et al.* 2015c), and some showed varying results across individuals (in polar bears: Kelly *et al.* 2015). By necessity, these studies typically correlate the number of visitors during an observation session with changes in behavioural and/or hormonal indicators of stress (e.g. Mallapur *et al.* 2005; Amrein *et al.* 2014; Kelly *et al.* 2015; Sherwen *et al.* 2015c; Krebs *et al.* 2023). While the studies were productive in illustrating associations between visitors and changes in welfare measures, a pertinent critique is that husbandry events and weather might also explain these changes (Goodenough *et al.* 2019; Rose *et al.* 2020). For example, in ring-tailed lemurs (*Lemur catta*), weather variables accounted for a higher proportion of variability in stress indicators than visitor presence (Goodenough *et al.* 2019). Similarly, a relationship between keeper interactions and agonistic behaviours in chimpanzees and gorillas has been found (Wood 1998). In another study, activity levels of captive felids attracted visitors to the exhibit (Margulis *et al.* 2003). If highly anticipated feeding schedules increased activity/anticipatory behaviour frequencies (Bassett & Buchanan-Smith 2007), then visitor presence was not the cause of the increase in stress indicator but rather an effect of the animal’s behaviour. Experimental manipulations and testing outside of the *status quo* of the animals’ experiences of visitors could address these concerns.

Manipulating different aspects of animals’ experiences of visitors has revealed interesting types of effects of visitors on the welfare of captive species. For example, Chamove *et al.* (1988) manipulated visual effects of visitors on ring-tailed lemurs, Diana monkeys (*Cercopithecus diana*), and cotton-top tamarins (*Sanguinus oedipus*) by asking visitors to crouch upon approach to a window which resulted in fewer instances of agonistic behaviours, inactivity, and grooming when compared to when visitors were upright. Similar manipulations have been undertaken for visibility and choice for escape (visual barriers in black-capped capuchins [*Sapajus apella*]: Sherwen *et al.* 2015a and in western lowland gorillas: O’Malley *et al.* 2021), noise (asking visitors to make noise or stay quiet in Sumatran orangutans [*Pongo abelii*]: Birke 2002), and presence (randomised exhibit closure in little penguins: Sherwen *et al.* 2015c).

Visitor presence effects are difficult to manipulate experimentally due to zoos’ reliance upon the presence of visitors. However, the lockdowns due to the COVID-19 pandemic forced zoos to close for weeks or months at a time. This prolonged absence was a change in *status quo*, which meant that visitor presence and absence could be studied while routine factors continued regardless of visitor presence or absence. In effect this was a natural experiment akin to systematic closures in experimental studies. The lockdown dissociated previously co-occurring factors such as visitor density, feeding events, and time of the day, allowing us to understand the factor of visitor presence separately. While quasi-experimental designs like a lockdown cannot demonstrate causality, these lockdown studies can capture the effect of a rare prolonged human absence and provide comparative findings for experimental and observational visitor effect studies alike.

A majority of the visitor effect studies during COVID-19 lockdowns, particularly those on great apes, showed varying changes across species (e.g. Bernstein-Kurtycz *et al.* 2021; Jones *et al.* 2021; Edes *et al.* 2022; Finch *et al.* 2022; Frost *et al.* 2022; Masman *et al.* 2022; Salak & Cloutier Barbour 2022; Williams *et al.* 2022). For example, gorillas at St Louis Zoo in the US, showed an initial increase in preference for areas away from visitors that diminished over the following months (Edes *et al.* 2022). The authors suggest that this space preference among the gorillas could be related to contextual variables; in their case it was the sun. They deemed the effect to be overall neutral despite the initial and fleeting negative effect on welfare (Edes *et al.* 2022). Meanwhile gorillas at Buffalo Zoo in the US, did not show substantial changes in their behaviours before or during lockdown (Masman *et al.* 2022). Meanwhile, gorillas at Twycross Zoo in the UK decreased resting and time spent with conspecifics which the authors considered to be a potentially negative effect (Williams *et al.* 2022). The chimpanzees and bonobos from the same study appeared to have the opposite effect whereby chimpanzees increased time interacting with enrichment and bonobos spent more time with conspecifics which were deemed positive (Williams *et al.* 2022). None of the primate species they studied showed increases in the concentration of faecal glucocorticoid metabolites (Williams *et al.* 2022). In contrast to studies that found negative effects in gorillas and chimpanzees, the mixed effects found in these studies suggest that effects could vary not only across species but also across institutions.

In comparison to other great ape species, there are relatively fewer studies of visitor effects in orangutans (Birke 2002; Amrein *et al.* 2014; Bloomfield *et al.* 2015). This is surprising given that Sumatran orangutans are critically endangered great apes and are projected to decrease to only 4,500 individuals left in the wild by 2030 (Wich *et al.* 2016), meaning that at some point there may be more orangutans living in captive settings than in the wild. As primates, their popularity with visitors in zoos (Whitworth 2012) could put them at risk of incurring the negative effects of visitor presence as a result of increased exposure. It is therefore ethically and morally justifiable (Hutchins *et al.* 2003) to ensure that the presence of visitors is conducive to their well-being in human care.

The effects of visitors that have been studied in orangutans varied, not only in the type of effect (e.g. visual, presence, noise etc), but also the way the visitor variable was manipulated (or not) and the direction of the effect on welfare (i.e. negative, positive or neutral; Hosey 2013). To examine the direction of visitor effects, scientists have often quantified fear or stress due to visitors using indicator behaviours such as gaze, infant-holding, or head covering, self-directed behaviours, or physiological measures like cortisol metabolites, (Amrein *et al.* 2014: [*P. pygmaeus*]: Birke 2002; Choo *et al.* 2011; Bloomfield *et al.* 2015). However, quantifying the direction of the effects based on the changes in indicators required interpretation which was different for each study and likely depended on how visitor presence was manipulated. For example, Birke (2002) measured the looking behaviour of orangutans in response to visitor noise. The orangutans performed more looking behaviours in the noisy condition than in the quiet condition, and this was interpreted as aggression towards visitors (Birke 2002). In contrast, Bloomfield and colleagues (2015) interpreted gaze as a *preference* for visitors. They found that orangutans oriented towards uncovered viewing windows more than covered windows (Bloomfield *et al.* 2015). Thus, in the context of an uncontrollable visitor noise as in Birke’s (2002) study, looking behaviour could have represented vigilance and thus a negative effect of a disruptive stimulus. Whereas when given full choice, looking and approach represented positive preference in Bloomfield *et al.*’s (2015) study. When visitor presence or behaviour was not manipulated but was simply observed naturalistically, the effects were more negative. Birke (2002) found that higher visitor numbers were associated with increased hiding and head covering which was interpreted as a negative effect in the non-experimental part of their study. In Bornean orangutans, Amrein *et al.* (2014) found a positive correlation between visitor numbers and both faecal glucocorticoid hormones and self-directed behaviours, which was interpreted as stressful and, thus, negative. It appears then that in the context of uncontrollable visitor presence, the effects of visitors on orangutan welfare may be negative. Other behavioural changes have also been observed with respect to an increase in the presence of active visitors. In a study of the effects of visitor behaviour, orangutans increased their begging behaviours when visitors were active rather than passively watching (Choo *et al.* 2011). The authors did not specifically attribute valence to this behavioural change (Choo *et al.* 2011). Nonetheless, given the complexity of visitor effects due to the contextual variables affecting the visitor effects observed, more research outside of the *status quo* would be helpful to compare with these findings. The COVID-19 lockdowns allowed the opportunity to study visitor effects on orangutan welfare in the context of prolonged visitor absence followed by a reintroduction of visitors that was completely uncontrolled by the animals.

The purpose of this study was to assess visitor presence effects on the welfare of Sumatran orangutans when COVID-19 lockdown measures were lifted at Toronto Zoo. In this small study, we build upon the findings of previous visitor effects studies on orangutans (Amrein *et al.* 2014 [*P. pygmaeus*]: Birke 2002; Choo *et al.* 2011; Bloomfield *et al.* 2015). We measured changes in multiple behavioural and physiological indicators of stress that have been used in previous studies. Specifically, we measured changes in self-directed behaviours, hiding, and faecal glucocorticoid metabolites. We supplemented these with other indicators of stress that have been used in other primates species such as agonistic behaviours (in olive baboons [*Papio anubis*]: Sapolsky & Ray 1989), agitated movement (in common marmosets [*Callithrix jacchus*]: Bassett *et al.* 2003), abnormal faecal consistency (in humans: Lemay *et al.* 2021), and some idiosyncratic object-directed manipulation behaviours (e.g. fabric tearing and head slamming on blankets) that we have previously observed among our sample. We also measured the amount of physical space used by the orangutans and their preference for each exhibit feature (Ross & Lukas 2006). Following the previous findings of visitor effects in orangutans, we hypothesised that there would be an increase in the frequency of behavioural stress indicators, concentration of faecal glucocorticoid metabolites, and proportion of abnormal faecal samples when visitors were reintroduced when compared to the lockdown. We also hypothesised that the amount of space used by the orangutans would decrease after the visitors were reintroduced, indicating that the animals preferred to sit in areas away from the visitors. Finally, we explored general changes in activity levels and foraging, as well as the effects of keeper presence.

## Materials and methods

### Subjects

We observed all six Sumatran orangutans housed at Toronto Zoo. Table 1 describes the orangutans in detail along with whom they were housed, and the number of hours they were observed across the phases of the study (lockdown vs visitor reintroduction). Each orangutan had a different housing condition (paired or single) either with a relative as with Jingga and Ramai or as a social/breeding pair like Sekali and Budi. Ramai was also occasionally paired with Budi on- and off-exhibit as a temporary breeding pair. With the exception of Puppe, all orangutans in this study were zoo-born; thus, have always been exposed to visitors when they were on-exhibit.

**Table 1.** All Sumatran orangutans (*Pongo abelii*; n = 6) housed at Toronto Zoo and included in the study

Individual	Age (years)	Sex	Paired/Solitary	Hours observed (lockdown)	Hours observed (visitor)
Jingga	15	Female	Paired (Ramai)	41.5	32.83
Ramai	36	Female	Paired (Jingga)	41.17	39.17
Budi	15	Male	Paired (Sekali)	52.5	46
Kembali	15	Male	Solitary	20.5	8
Sekali	28	Female	Paired (Budi)	54.5	43.83
Puppe	54	Female	Solitary	58	39.40

The orangutans were afforded free choice between going on-exhibit or staying in their off-exhibit space (i.e. holding). Once on-exhibit, the orangutans were kept there for either half the day or a full day (for Puppe only). This was in accordance with the orangutan protocol for Toronto Zoo whereby being locked outside remained consistent throughout the study. This meant, therefore, that orangutans were not observed for an equal number of hours. This was especially the case with Kembali, who was observed less than his conspecifics due to his refusal to stay on-exhibit. He was thus provided open access until he decided to stay in the exhibit, at which point he was locked in. The reason for his reluctance was not apparent. However, throughout the study, breeding regularly occurred in the holding between the flanged male Budi and Sekali or Ramai. The keeping staff suggested that this was one of the reasons why he chose to stay in the holding.

### Behavioural sampling

The observation sessions began on 3 May 2021 and ended on 17 August 2021. We used the ethogram in Table 2 to code the behaviours. Behaviours were sampled using point-scan sampling (Altmann 1974) with 10-min intervals for state behaviours. As the orangutans were housed in pairs or individually, the focal animal was whoever was present on-exhibit or were simultaneously observed. In the case of the paired orangutans, we scanned them in sequential order. This order varied across observation schedules but not within observation schedules. We collected *ad libitum/*all-occurences samples to supplement the scan samples. We initially used *ad libitum* sampling (Altmann 1974) for behaviours that were rarely observed and typically short in duration (i.e. < 1 min per bout), particularly, agitated movement, agonistic behaviour, object-directed displacement, and self-directed behaviours occurred. The *ad libitum* data collection proceeded as follows: as new behaviours occurred that we had not defined in the ethogram, we noted and, when possible, described all occurrences of these behaviours including the time, and location. As different forms of the same behaviour appeared (e.g. rough scratching the back of nape vs rough scratching of the abdomen), they were assigned to the same category. These data were not discarded and eventually added to the all-occurrences sampling during analysis. This was done because the all-occurrences sampling method did not differ from the *ad libitum* sampling, with the exception of having to regroup the behaviours. Starting on 13 May 2021 we sampled all occurrences (Altmann 1974) of event behaviours instead of *ad libitum* sampling, because at this point most of the behaviours were already being captured by the categories. We classified behaviours previously sampled using *ad libitum* sampling as agitated movement, agonistic behaviour, object-directed displacement, or self-directed behaviours. Any new behaviours that appeared from then were described in the notes and either grouped within one of these categories or given its own category. We also concurrently recorded the presence of keepers using the all-occurrences sampling method. We defined keeper presence as the presence of a keeper at the public area or in the keeper cage. The *ad libitum/*all-occurrences sampling were collected concurrently with the point-scan sampling methods such that every 10 min a state behaviour was sampled and the rare behaviours were sampled as they occurred. When a rare behaviour occurred at a defined time-point for a scan, they were sampled both in the point-scan and the all-occurrences sampling. We did this because the point-scan and all occurrences were analysed differently: as percent of scans and per minute rates, respectively. The state behaviours were summarised as relative frequencies and event behaviours as rates per minute. We summarised the relative frequencies and rates between the phases across individuals and observation sessions. The statistical units for analyses were each observation session.

**Table 2.** Ethogram of behaviours of the Sumatran orangutans (*Pongo abelii*; n = 6) included in the study

Behaviour type	Category	Code	Description
State	Foraging	F	Consumption of plant matter (e.g. leaves; soft vine barks; soft stalks, round fleshy parts). Marked by insertion of plant matter into the mouth with the use of the hands. It starts with the use of the hands to pick plant matter from a bunch or pile, to pick apart plant matter and to break plant matter into small pieces. The hands or sticks are then used to bring plant matter into the mouth. This is followed by chewing (i.e. open and close movement of the jaw while the plant matter is either partially or fully in the mouth). This is culminated by swallowing; that is, the plant matter is no longer in the mouth nor outside the animal and the animal moves to get more. The bout stops when there is a pause in the behaviour > 3 s or another behaviour is performed.
State	Locomotion	L	The orangutan moves with the use of limbs from one point in the exhibit to the next point at least within a metre away from the origin. The orangutan may end up in the same location as the origin, but along the path should have gone at least a metre away from the origin. The orangutan may be locomoting bipedally or quadrupedally on plane surfaces like platforms and the ground. If the orangutan is on climbing structures but is supported by all 4 limbs, the movement is classified as locomotion.
Event	Object play	OP	Repetitive manipulation and inspection (visual and/or tactile) of inedible objects which are not part of another individual’s body. The individual is visibly engaged (i.e. the facial/head orientation is on the object being manipulated). Inspection or manipulation is done by mouth, hands, or feet). Movement may appear like other behavioural categories but the size/speed of movements of limbs are exaggerated.
Event	Fiddling	FD	Slow and repetitive manipulation of an object with *no apparent purpose or engagement* (i.e. the orangutan may appear like staring in space and the gaze is not directed towards the moving object). Manipulation may be subtle repetitive finger movements along the object being manipulated
State	Inactive	I	The animal stays in the same spot or turns around but does not go beyond a metre from origin. The animal is not engaged in self-directed behaviours, defaecation, urination, foraging, hiding, defaecation, or urination or scanning behaviours or social interaction. The animal may be lying prone, supine, sideways, upright sitting, or quadrupedal but stationary.
Event	Affiliative	AF	The animal engages in social interactions with another individual such as allogrooming, begging for food, food sharing, hugging, tolerance. Behaviours would appear to maintain bond as seen by maintenance of close proximity. These behaviours do not have audible vocalisations or vigorous movements.
Event	Agonistic	AG	Social interactions with individuals where distance from each other is the outcome unless there is a physical confrontation or fight. The animal may be engaged in rejection of begging, or avoidance, or vigorously grabbing food from the grasp of the receiver of the interaction. Characterised by vigorous movements towards or away from the other individual.
Event	Keeper-directed	KD	Staring, following, locomoting towards the keeper, or obtaining food from the keeper. Attention/head orientation must be placed on the keeper. The keeper should be visible around the perimeter of the exhibit or in the keeper’s cage.
Event	Guest-directed	GD	Staring, following, or moving towards the guests. Attention must be placed on the guest. Volunteers (humans in white shirts and beige trousers) are considered guests.
Event	Self-directed	GR	Inspection of hair, body or mouth with hands, feet, mouth or with the use of objects such as browse, sticks or enrichment. The body part being inspected is prod repeatedly by any of the above-mentioned implements. The animal may scratch, squeeze, poke or pinch the body part being inspected. Attention/gaze does not have to be on the body part.
Event	Hiding^a^	H	The animal covers itself with a blanket, a leaf, or goes in the bucket such that only a portion of the head is visible for scanning.
Event	Urinate	U	Marked by the presence of darker wet spot on the floor. Urine flows from the hind of the orangutan. The orangutan may be hanging on climbing structures using any combination of limbs or may be sitting at the edge of the moat, platform, or on a bar with the hind facing where the urine would land.
Event	Object manipulation	OM	Moving objects with limbs or the mouth from one point in the enclosure to the other point. There is a very clear purpose that usually stops once the purpose has been achieved.
Event	Scanning	SC	The animal makes a short sweeping movement of the head, and the eyes stay forward following the gaze. The attention must be on anything outside the exhibit. The animal may be sitting on the floor or bipedally/quadrupedally locomoting towards a window or the edge of the exhibit.
Event	Patrolling	PT	The animal follows a repeated path around a portion or the entirety of the perimeter of the exhibit. The animal seems vigilant with repeated scans as movement happens.
Event	Defaecate	D	Marked by the presence of faecal matter on the floor. Faeces drops from the hind of the orangutan. The orangutan may be hanging on climbing structures using any combination of limbs or may be sitting at the edge of the moat, platform, or on a bar with the hind facing where the faeces would land. The orangutan may also reach around such that the faeces would land on the palm and the orangutan would drop the collected faeces on the floor. The orangutan may also gradually orient the upper body from an upright sitting position to a more acute prone posture.
Event	Agitated movement	AM	Locomotion that is fast, with fast scanning of surroundings, may or may not stop at a destination. Usually follows a loud noise. Brachiation along the bars is hasty and may involve short airtime. Scans towards the keeper’s kitchen or the entrance to the exhibit may be possible. Recoil is observed when hands or feet touch climbing structures or platforms.
Event	Idiosyncratic object-directed behaviour	AB	Objects are touched with the use of the hand or other body parts. The contact may be brief or prolonged. Includes head slamming onto blankets where recoil happens after contact. May also be forceful fabric tearing wherein the orangutan uses both hands to pull fabric apart in opposite directions swiftly and with recoil.

The ethogram of behaviours was non-exhaustive but was written using behaviours performed by the orangutans housed at Toronto Zoo and included in this study (n = 6). All the behaviours were functional and mutually exclusive. State behaviours were sampled using the point-scan sampling and event behaviours were sampled using *ad libitum/*all occurrences sampling. Event behaviours that occurred during a point-scan time-point were sampled for both. This rarely happened during the study.

a Hiding was distinguished from body wrapping (considered inactive) by the covering of the head (after Birke 2002). Proportion of the head visible usually are the eyes or the top of the head if the orangutan was crouched down in the bucket.

Observation sessions lasted approximately 2 h in the morning (0830–1230h) and 2 h in the afternoon (1240–1600h). The orangutans were on-exhibit for 4 h a day and were then replaced by a different orangutan at 1230h. Thus, there were two observation sessions per day to cover the afternoon and the morning sessions. All of these observation sessions took place on weekdays. With the exception of Puppe who stayed on-exhibit all day, all the orangutans had one observation session per day. The orangutans were typically visible during an entire session due to the design of the exhibit and due to being kept in. Orangutans were able to ‘hide’ in the bucket or wrap themselves in enrichment covering the entire body and the head except for a small portion left for the eyes. Since we considered ‘hiding’ as a separate behavioural category, these data-points were not removed from the analysis. However, we removed data-points for which access was left open at the beginning of the session. Initially, these were included in the analysis. However, when we reduced the all-occurrences sampling duration and the number of scan samples to the duration the animal was on-exhibit, we did not find any differences in the significance level nor the direction of the effects we observed. We thus report the data for which the animals were on-exhibit.

Originally, the study had three phases (lockdown, limited visitors, and fully opened). However, due to the short time-period with limited visitors (5 July–16 July), there was insufficient time to gain enough sample hours for all the orangutans. After comparing the behavioural data from the two visitor phases to show that they were not significantly different from each other, we combined the limited visitor phase with the fully opened phase. Therefore, the sampling for the lockdown phase started on 3 May 2021 and ended on 2 July 2021. Meanwhile, the fully opened phase started on 5 July 2021 to 17 August 2021. It is important to note that the visitor counts fluctuated for each scan sample during the visitor introduction phase, and thus some scans involved more visitors than others. The mean (± SD) number of visitors within 1 m of the exhibit boundaries across each scan sample during the visitor introduction phase was 12.95 (± 11.52). Other than to show how many visitors were present during each scan, we did not include visitor count in the models because the number of visitors was related to the phase. This made teasing apart the effects of visitor count and the phase of the study difficult.

### Enclosure use

**Figure 1.**
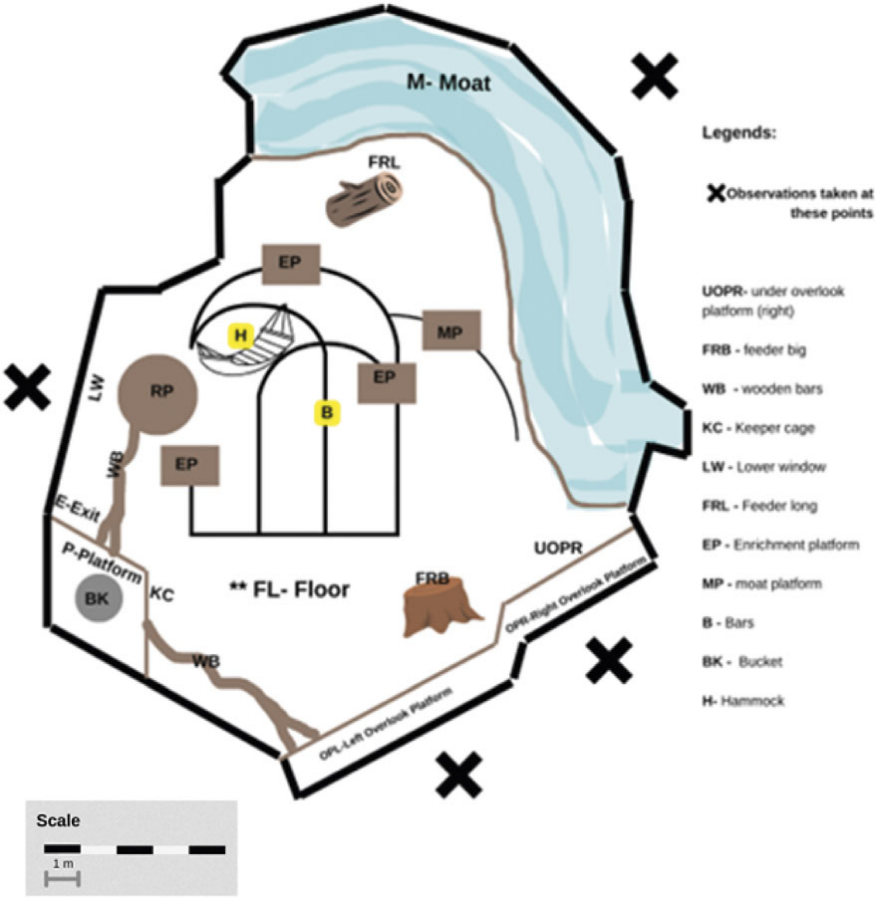
Toronto Zoo Sumatran orangutan (*Pongo abelii*) indoor exhibit schematic diagram. Diagram shows the location of specific exhibit features which are labelled with their codes. FL: the whole floor except other defined areas.

Figure 1 shows a schematic diagram of the orangutan enclosure, the relevant features within the exhibit, and the public area where the observations were taken. We sampled choice of enclosure areas concurrent with the behavioural point-scans. The selectivity of enclosure areas were measured using the modified spread of participation index (SPI) (Ross *et al.* 2011). Expected frequencies using the modified SPI equation typically use the estimated proportion of the enclosure area. However, because our study species used the available vertical climbing structures, we utilised the proportion of volume instead of area, after Ross *et al.* (2009). While Ross *et al*. (2009) used 1 m^3^ divisions akin to the original SPI equation which uses 1 m^2^ squared areas, we divided our exhibit by exhibit features. Nonetheless, because the modified SPI equation also used proportions, which is a dimensionless index, we were able to generate expected frequencies with the proportion of volume instead of area. Here, we estimated the proportion of the volume occupied by each vertical structure to produce the expected frequencies. We approximated the volume for each exhibit feature using the closest standard volume formula for the structure (e.g. a cone for the climbing structure). For flat surfaces such as platforms, the height was set arbitrarily to 1.5 m to account for the height orangutans could theoretically occupy were they to stand bipedally with arms raised as was occasionally the case when they were seeking to reach enrichment. We summed the total volume for all exhibit features and divided each exhibit feature volume by the total volume to produce the breakdown in proportion of volume occupied by each exhibit feature (Table 3). We used the sum of the frequencies of scans an orangutan was seen in each exhibit feature as the observed frequency. The modified SPI equation yields a score between 0–1 (0 being equal use and 1 being unequal use). Each orangutan had an SPI score for each phase. These SPI scores were the statistical units for this analysis. We also noted the most used location for each phase to ascertain what location was preferred the most (see Table 3).

**Table 3.** The use of exhibit features by the Sumatran orangutans (*Pongo abelii*; n = 6) included in the study at Toronto Zoo

			Scans (%) where exhibit was used by at least one orangutan	
Exhibit feature	Code	Estimated % of total space (volume) available taken up by exhibit	Lockdown	Visitor phase
Floor	FL	51.8%	3.3%	4%
Metal Bars	B	12.9%	6.4%	8.3%
**Back platform**	**P**	**3.9%**	**39.8%**	**25.3%**
Moat Beach	M	5.2%	0.7%	0.4%
Round platform	RP	4.2%	3.7%	3.1%
Overlook platform (Left)	OPL	3.5%	5.9%	21.6%
**Under overlook platform (Left)**	**UOPL**	**3.5%**	**0%**	**0.1%**
Lower window	LW	3.4%	10.4%	5.4%
Keeper cage	KC	2.2%	4.2%	4.9%
Feeder stump (Long)	FRL	2%	0.3%	1.8%
Overlook platform (Right)	OPR	1.7 %	5%	7.4%
**Under overlook platform (Right)**	**UOPR**	**1.7%**	**1.3%**	**0.7%**
**Hammock**	**H**	**1.3%**	**1.4%**	**2.9%**
**Bucket**	**BK**	**0.7%**	**7.6%**	**3.6%**
**Enrichment platform**	**EP**	**0.9%**	**5.6%**	**5.1%**
**Exit**	**EXIT**	**0.4%**	**0.3%**	**1.1%**
Wooden Bars	WB	0.4%	0.9%	1.0%
Moat platform	MP	0.2%	0.3%	0.2%
Feeder Stump (Big)	FRB	0.2%	2.3%	1.9%
Total estimated usable volume:		343.8 m^3^	Scans; n = 1,565	Scans; n = 1,247

Items shown in bold represent features away from visitor areas.

### Faecal consistency

Between 8 June and 3 August 2021, the keeping staff collected daily faecal samples before 1000h for all orangutans whether they were on-exhibit or not. Not all orangutans, however, produced faeces daily. The orangutans were housed in individual holding rooms in the off-exhibit space; hence the care staff were able to trace the faeces to the individual. When the mother-daughter pair were housed together in the off-exhibit space, their faeces were combined and were thus excluded from analysis. Faeces were collected in resealable plastic bags then labelled with the date, time, and individual identity prior to being frozen for storage. Frozen samples were transported to the Glendon Primate Behavioural Endocrinology Lab (York University) and stored at –20°C until ready to be thawed for faecal consistency and hormone extraction. Within an hour of thawing, the faecal consistency of each sample was rated using the 7-point Bristol stool scale (Lewis & Heaton 1997). Per the original classification suggested by Lewis and Heaton (1997), we categorised faecal consistency ratings of 3 and 4 as normal (scored as 0) and ratings less than 3 and greater than 4 as abnormal (scored as 1). These scores are indicative of diarrhoea (> 4) or constipation (< 3) in human faecal samples (Lewis & Heaton 1997).

### Faecal glucocorticoid metabolites

After being rated for consistency, faeces were manually homogenised in the plastic bags, ensuring that any accumulated moisture was mixed back into the faecal matter. A 0.5-g aliquot of each wet faecal sample was used to determine dry weight and another for hormone extraction and subsequent enzyme immunoassay (EIA). To determine dry weight, we placed the 0.5 g of faeces onto aluminum weigh boats and they were dried in an oven at 70°C for 4 h. Once drying was complete, samples were reweighed and the proportion of dry faeces weight calculated. We extracted faecal hormone metabolites by suspending the 0.50 g of wet faeces in 5 ml of an 80% methanol solution (Palme 2005), shaking on a multitube vortex for 30 min, and centrifuging at 3,500 rpm for 10 min. This resulted in a 10-fold dilution of hormone-methanol extracts, all of which were stored at –20°C until ready for glucocorticoid quantification.

We followed the manufacturers’ instructions for the Cortisol Enzyme Immunoassay Kit (Arbor Assays K003-H5W, Arbor Assay, Michigan, USA), with a 75-uL aliquot of the hormone-methanol extract. Previous research at Toronto Zoo validated the use of this cortisol assay for orangutan faecal samples (Berkvens 2012). All samples and standard curves were run in duplicate. Optical density was measured for each plate at 450 nm on a Synergy LX spectrophotometer (Biotek Instruments, Ltd. Vermont, USA), and then converted the returned values to ng g^–1^ of wet faecal weight using the associated Gen5 (v.3.11) software. To calculate the dry weight hormone concentrations (ng g^–1^), we divided the wet weight hormone concentrations (ng g^–1^) by the proportion of dry faeces weight. Using hormone concentration value for dry weight accounts for the effects of faecal composition on the concentration of cortisol metabolites (Goymann 2012).

To test for parallelism, serial dilutions in EIA buffer of the orangutan hormone extract were compared against a 9-point standard curve (62.5–16,000 pg mL^–1^). Visual examination shows a high degree of parallelism between the standard curves and the serially diluted samples. The mean intra- and inter-assay coefficients of variation for faecal glucocorticoids were 15.9 and 10.9%, respectively.

### Statistical analysis

We compared the measures between the lockdown phase (3 May 2021–4 July 2021), when zero visitors were allowed in the pavilion, and the visitor phase (5 July 2021–30 August 2021) when a maximum of 200 people were allowed in the pavilion. The alpha level for all analyses was 𝛼 = 0.05. To assess the effect sizes, we reported the 95% confidence interval for the parameter estimate of concern. All statistical analyses were conducted using R (R Core Team 2021).

#### Behaviour models

Despite the ethogram (Table 2) containing most of the behaviours the Toronto Zoo Orangutans performed, we only analysed those behaviours relevant to our hypothesis. We fitted linear mixed effects models using the ‘lme4’ package in R (Bates *et al.* 2015) to analyse agitated movement, self-directed behaviours, foraging, inactivity. All mixed models used the *individual animal ID* as the random effect predictor and the rate or percent of scans of a certain behaviour as the response variable. The full model included *phase* and *keeper presence rate* as fixed effect predictors in order to control for the effects of keeper presence on behaviour. We then used likelihood-ratio tests to compare the full model to a simpler model with only *keeper presence rate* as a fixed effect predictor. This tests whether the addition of *phase* to the model significantly explained the variability in a behaviour over and above what was explained by the individual differences and the effect of *keeper presence rate.* Meanwhile, we analysed changes in agonistic behaviours and idiosyncratic object directed behaviours (fabric tearing and head slamming) within each individual using multiple linear regressions with the *phase* and *keeper presence rate* as the predictor. These behaviours were analysed separately for each individual for two reasons: (1) not all of the individuals were housed socially and when they were housed socially, they were paired with different individuals; and (2) only two individuals performed idiosyncratic object behaviours and each of them performed a different kind to the other. Combining the analyses for agonistic behaviours into one mixed effect model would violate independence of errors assumptions due to the paired housing. Meanwhile combining the idiosyncratic behaviours into one mixed effect model would result in a very small sample size (n = 2).

#### Space use

We fitted a linear mixed effect model with the SPI as the response variable, the *individual animal ID* as the random effect predictor and the *phase* as the fixed effect predictor. The SPIs were calculated over an entire phase for each orangutan; thus, the keeper presence rate could not be added into the model. Therefore, we performed a likelihood ratio test between a model with a phase and an unconditional model with only the random effect predictor to produce an omnibus test statistic to assess the fit of the model.

#### Faecal measures

We fitted models for faecal consistency and for faecal glucocorticoid concentration, both of which included the *individual animal ID* as the random effect predictor. A generalised linear mixed effects model was fitted with a logit-link function to account for the variability in the incidence of diarrhoea and constipation in relation to the phase of the study. The generalised linear mixed effect model was used for faecal consistency because the response variable was binary (i.e. normal vs abnormal). We tested whether the addition of *phase* as a predictor explained a significant proportion of the variability in the log-odds of the incidence of diarrhoea or constipation (i.e. abnormal faeces). We did not use an interaction model (being on-exhibit the day before and phase) to analyse the effects of the phases on faecal consistency. Instead, we fitted a model with the *phase* and *presence on-exhibit* as fixed effect predictors without an interaction. The intestinal transit time for orangutans varies (in *P. pygmaeus* (Caton *et al.* 1999), which meant that non-exhibit days could also yield abnormal faecal samples due to stress. By raising Euler’s number, *e*, to the estimated coefficients from the model, we estimated the odds ratios of the likelihood of an abnormal faecal consistency score between the levels of the predictor variables.

Lastly, for the faecal glucocorticoid model, we modelled the interaction of being on-exhibit or not and the phase of the study. Using a likelihood ratio test, we tested whether the addition of the interaction between *being on-exhibit the day before* and the *phase* to the model significantly explained the variability in the *faecal glucocorticoid concentration* over and above what was explained separately by the *phase* and the *presence on-exhibit the day before.* That is, the fixed effect predictor variables were *phase* and *presence or absence on-exhibit*, whereas the response variable was *faecal glucocorticoid concentration* (ng g^–1^). We reported the estimates of each predictor variable to understand the direction and significance of the effects of each predictor variable. We report all the final models in Table 4.

**Table 4.** Mean differences between lockdown and visitor reintroduction phase of each behavioural welfare indicator in the study of Sumatran orangutans (*Pongo abelii*; n = 6) at Toronto Zoo

Behavioural indicator	Mean difference	*t*-value	df	P-value
Self-directed behaviours (% of scans)	–1.31	–1.85	246.83	0.07
**Self-directed behaviours (rate per min)**	**0.01**	**4.05**	**263.9**	***<* 0.001** ^a^
Agitated movement (rate per min)	0.00006	0.103	218.3	0.92
Agitated movement (% of scans)	0.03	0.17	247.31	0.87
Agonistic behaviour (Jingga; rate per min)	0.005	1.901	41	0.06
Agonistic behaviour (Sekali; rate per min)	–0.002	–0.95	60	0.35
Agonistic behaviour (Budi; rate per min)	0.003	0.79	50	0.44
Agonistic behaviour (Ramai; rate per min)	0.002	0.725	44	0.47
Fabric tearing (Sekali: rate per min)	0.0006	0.46	60	0.65
Head slamming (Puppe: rate per min)	–0.0001	–0.04	43	0.97

Mean difference (Visitor–Lockdown) between the lockdown phase and the visitor reintroduction phase in each behavioural indicator observed holding keeper presence rate constant. If individual orangutan name was indicated, the mean difference was estimated for each orangutan using ordinary least squares regression with phase and keeper presence rate as predictors. Otherwise, models were estimated using linear mixed effects models with the structure: stress indicator = phase+keeper presence rate + (1|animal ID). Items in bold were statistically significant at alpha = 0.05.

a Whilst statistically significant, the mean difference between visitor phase and the lockdown was practically negligible (0.01 higher per min).

### Ethical statement

All observations were carried out in the public areas of the zoos, approved by the Toronto Zoo Animal Welfare Committee and followed the American Society of Primatologists Principles for the Ethical Treatment of Non-Human Primates. All observations were made under Canadian Council on Animal Care (CCAC) guidelines. Toronto Zoo is a CCAC-accredited institution and the zero-visitor phase was implemented by the zoo as part of their pandemic control strategy. This study was observational and conducted as part of the long-term behavioural monitoring of this species implemented by Toronto Zoo; therefore, it did not change the lives of the orangutans we studied. However, care was taken to limit interactions between the observer and the animals by maintaining at least a 1-m distance between the viewing and the observer. Except for data on presence (i.e. count or frequency), no detailed data on visitors or keepers were collected.

## Results

### Exhibit space use

**Figure 2.**
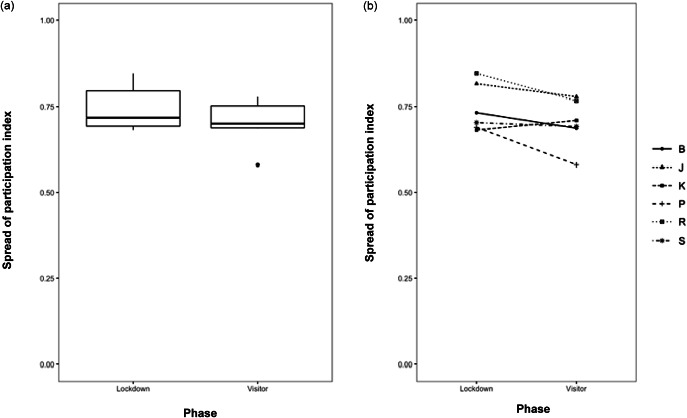
Spread of participation index (SPI) scores for Toronto Zoo orangutans (*Pongo abelii*) (n = 6) during lockdown and visitor study phases for (a) all orangutans (n = 6) and (b) individual orangutans (B = Budi, J = Jingga, K = Kembali, P = Puppe, R = Ramai, S = Sekali). The SPI measures selectivity of exhibit area and ranges from 0 (equal use of areas) to 1 (exclusive preference for one area).

As seen in Figures 2(a) and (b), the reintroduction of visitors after the lockdown measures did not significantly affect the amount of space in the habitat that the orangutans used. Whereas the model significantly predicted the variability in SPI scores (*Likelihood ratio test*: χ^2^[1] = 3.93; *P =* 0.04743), the effect of the phase was not significant. The orangutans did not significantly change their use of specific areas of the exhibit in response to the reintroduction of visitors (*t*[5] *=* –2.51; *P =* 0.08) in their pavilion. The breakdown of the use of specific exhibit areas across the phases of this study in Table 3 shows that orangutans did not avoid areas close to visitors when visitors were reintroduced. The orangutans did not substantially increase the use of areas such as the exit, the back platform, under the overlook platform, and hammock, enrichment platforms, and the bucket. However, the orangutans were already very selective regarding what areas of their habitat they used. The orangutans spent a majority of the time (38% during the lockdown, 25% during the visitor phase) at the back platform (Table 3). This area is close not only to the keepers’ entry point to the exhibit area for providing food and enrichment, but also to the exit to the orangutan holding (off-exhibit space).

**Figure 3.**
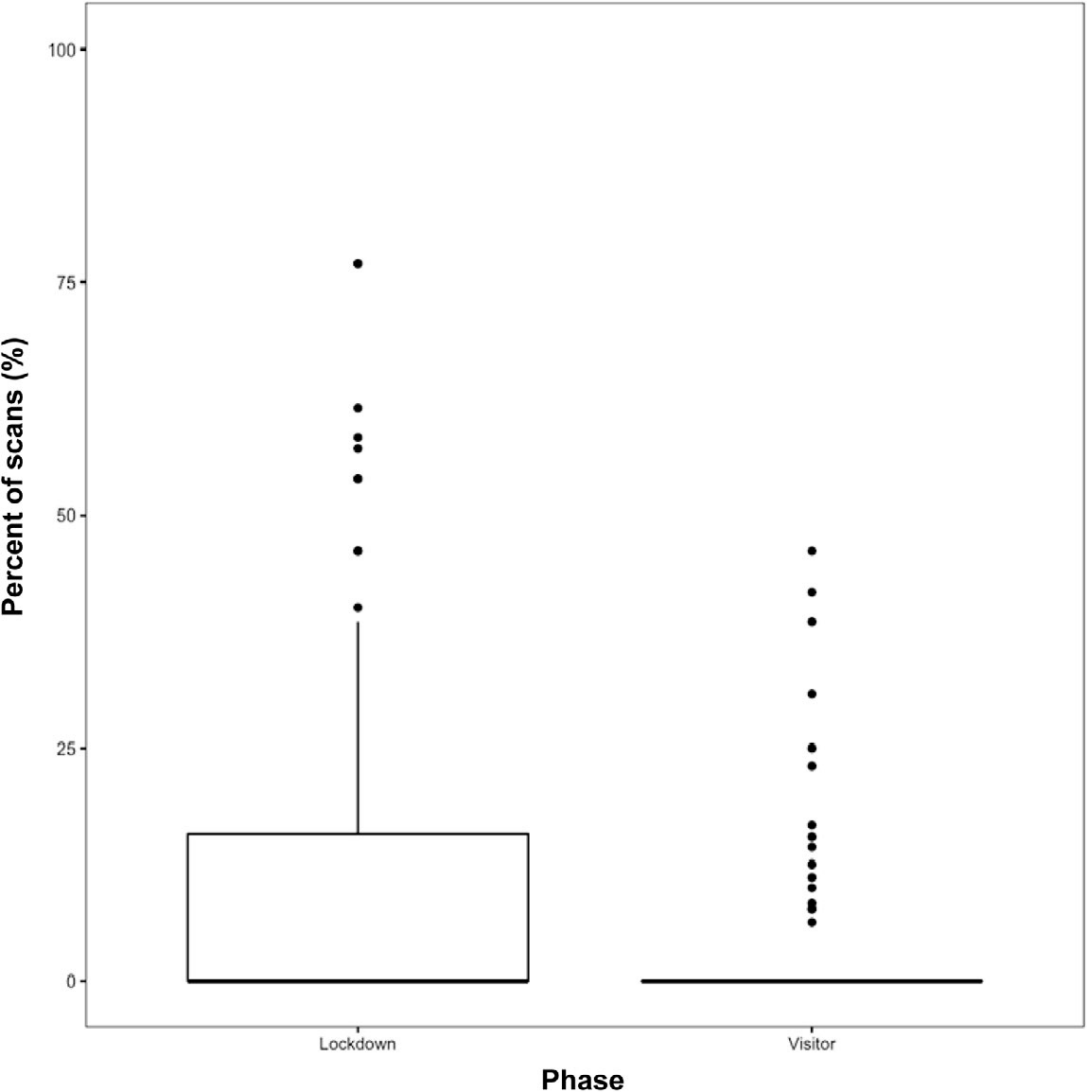
The percent of scans the orangutans (n = 6) were observed hiding across the phases of this study (lockdown vs visitor).

Meanwhile, the phase of the study significantly explained the variability in the proportion of scans the orangutans were seen hiding (*Likelihood ratio test*: χ^2^[1] = 17.542; *P* < 0.001). However, the orangutans did not increase hiding behaviours when visitors were reintroduced (Figure 3). In fact, when compared to the lockdown phase, the orangutans decreased their hiding significantly (*t*[246.59] *=* –4.255; *P <* 0.001, 95% CI [–11.98, –4.41]) when visitors were introduced. The rate of keeper presence did not predict the change in the proportion of scans the orangutans were hiding (*t*[247.63] = 0.73; *P* = 0.46).

### Foraging and inactivity

The phase did not explain a significant proportion of variability in inactivity (*Likelihood ratio test*: χ^2^[1] = 2.16; *P* = 0.14) or foraging (χ^2^[1] = 2.13; *P* = 0.14) above and beyond what was already explained by keeper presence and individual variability. There was no difference (*t*[247.40] *=* 1.46; *P =* 0.15, 95% CI [–1.04%, 7.06%]) between the mean (± SD) percent of scans the orangutans spent foraging during the lockdown phase (16.62 [±13.73]%) and the visitor phase (19.37 [± 19.05]%). Similarly, there was no difference (*t*[246.08] *=* 1.46; *P =* 0.15, 95%, CI [–1.32%, 9.19%]) between the mean (± SD) percent of scans the orangutans spent inactive during the lockdown (37.45 [± 22.98]%) and the visitor phase (43.13 [± 25.61]%). The rate of keeper presence did not predict the value of the percent of time foraging (*t*[249.42] *= –*10.79; *P =* 0.86, 95%, CI [–134.76%, 109.34%]) nor the percent of time inactive (*t*[249.42] *=* 56.21; *P =* 0.49, 95%, CI [–101.83%, 213.56%]).

### Behavioural indicators

To examine the amount of variance in the indicator of welfare explained by the reintroduction of visitors over and above what was explained by keeper presence and individual orangutan differences, likelihood ratio tests were performed. We then examined the mean differences across conditions to understand the direction and magnitude of the change. Table 4 summarises the mean differences of displacement behaviours between the two phases of the study controlling for keeper presence while Table 5 shows the estimated effect of keeper presence on the behavioural indicators holding the phase constant. We disentangle the effects of visitor reintroduction and keeper presence below.

**Table 5.** Estimated effect of the rate of keeper presence on each behavioural welfare indicator in the study of Sumatran orangutans (*Pongo abelii*; n = 6) at Toronto Zoo

Behavioural indicator	B	*t*-value	df	*P*-value
**Self-directed behaviours (% of scans)**	**60.16**	**2.83**	**247.79**	**0.01**
**Self-directed behaviours (rate per minute)**	**0.25**	**3.67**	**263.9**	***<* 0.001**
Agitated movement (rate per minute)	0.02	1.17	219.4	0.25
Agitated movement (% of scans)	–3.31	–0.55	249.9	0.58
Agonistic behaviour (Jingga; rate per minute)	–0.06	–0.80	41	0.43
Agonistic behaviour (Sekali; rate per minute)	0.03	0.55	60	0.58
Agonistic behaviour (Budi; rate per minute)	**0.27**	**2.37**	**50**	**0.02** ^a^
Agonistic behaviour (Ramai; Rate per minute)	–0.08	–1.33	44	0.19
Fabric tearing (Sekali: Rate per minute)	0.02	0.53	60	0.60
Head slamming (Puppe: Rate per minute)	0.08	0.90	43	0.37

Estimated increase on the rates or frequencies of behavioural indicators for a one unit increase in the rate of keeper presence holding reintroduction phase constant. If individual orangutan name was indicated, the mean difference was estimated for each orangutan using ordinary least squares regression with phase and keeper presence rate as predictors. Otherwise, models were estimated using linear mixed effects models with the structure: stress indicator = phase+keeper presence rate + (1|animal ID). Items in bold were statistically significant at alpha = 0.05.

a While the effect estimate was statistically significant, the overall model did not significantly predict variance in Budi’s agonistic behaviour rate.

#### Self-directed displacement behaviours

Phase did not explain a significant proportion of variability in the percent of scans of self-directed behaviours above and beyond what was already explained by keeper presence and individual orangutan differences (*Likelihood ratio test*: χ*^2^*[1] = 3.44; *P =* 0.06). The orangutans did not significantly increase the amount of time they spent performing self-directed behaviours (*B =* –1.31, *t*[246.83]*=* –1.85; *P =* 0.07, 95% CI [–2, 0.07]). Since displacement behaviours were rarely seen, we also analysed the increase of frequencies in these behaviours. Adding phase to the model added a significant proportion of variability in rates of self-directed behaviour above and beyond what was explained by keeper presence and individual orangutan differences (*Likelihood ratio test*: χ*^2^*[1] = 16.12; *P <* 0.001). The orangutans increased their rates of self-directed behaviours by 0.01 events per min during the visitor introduction phase when compared to the lockdown phase (*t*[263.9] *=* 4.05; *P* < 0.001). Practically, the effect size was too small (95% CI [0.006, 0.02]) for this to be a meaningful difference. This is equivalent to an increase in rate of 1 bout per 100 min. Meanwhile, for every unit increase in rate of keeper presence, the rate of self-directed behaviour increases by 0.25 (*t*[265.4] *=* 3.67; *P* < 0.001, 95% CI [0.11, 0.37]).

#### Object-directed behaviours

Only two (Puppe and Sekali) of the six orangutans performed idiosyncratic behaviours directed towards objects (fabric tearing for Sekali and head slamming into a pile of blankets for Puppe). For both of these individuals, the frequencies of these behaviours were even rarer than self-directed behaviours. When the rates of these behaviours were regressed on the phase and the keeper presence, the proportion of variability in the response variable explained by the predictors was not significant (*F-*test; Sekali: *F*_2,60_ = 0.26; *P* = 0.77; Puppe*: F*_2,43_ = 0.40; *P* = 0.67). Neither Puppe (*B =* –0.0001, *t*[43] = –0.04; *P* = 0.97, 95% CI [–0.0045, 0.004]) nor Sekali (*B =* 0.0006, *t*[60] = 0.46; *P* = 0.65, 95% CI [–0.0045, 0.004]), respectively, showed any change in the rates of these behaviours when visitors were reintroduced.

#### Agonistic behaviours

Regressing rates of agonistic behaviours on phase and keeper presence rate did not significantly predict variability in rates of agonistic behaviours performed by each orangutan (*Likelihood ratio test*: Sekali: *F*_2,60_ = 0.57; *P* = 0.57; Budi: *F*_2,50_ = 3.14; *P* = 0.052; Ramai: *F*_2,44_ = 1; *P* = 0.38; Jingga: *F*_2,41_ = 1.969; *P* = 0.15). Sekali’s agonistic behaviour rate did not significantly change towards Budi (*B* = –0.002, *t*[60] = –0.95; *P* = 0.35, 95% CI [–0.006, 0.002]), and Budi did not significantly increase his rate of agonistic behaviours towards Sekali when visitors were reintroduced (*B =* 0.003, *t*[50] = 0.79; *P* = 0.44, 95% CI [–0.006, 0.002]). Ramai’s agonistic behaviours towards Jingga did not change when visitors were reintroduced (*B =* 0.002, *t*[44] = 0.725; *P* = 0.47, 95% CI [–0.003, 0.006]). Jingga’s agonistic behaviours towards Ramai did not significantly change when visitors were reintroduced (*B =* 0.005, *t*[41] *=* 1.901; *P =* 0.06, 95% CI [–0.0003, 0.009]). We pre-emptively watched for agonistic behaviours such as biting, forcing food out of another orangutan’s mouth or grasp, rapid locomotion away from another orangutan, and displays. Nonetheless, the only agonistic behaviours we observed involved an orangutan grabbing food from the other and the recipient of this action locomoting rapidly away from the initiator of the action.

#### Agitated movement

Puppe was excluded from these analyses due to her advanced age and slow movement. Adding phase as a predictor variable did not explain a significant proportion of variability in percent of scans of agitated movement over and above what was already explained by keeper presence and individual orangutan variability (*Likelihood ratio test:* χ*^2^*[1] = 0.03; *P =* 0.86). The orangutans did not significantly change the amount of time spent in the state of agitated movements (*B* = 0.03, *t*[247.31] = 0.17; *P* = 0.87, 95% CI [–0.36, 0.43]). Similarly, phase did not explain variability in rates of agitated movement above and beyond what was already explained by keeper presence and individual orangutan variability (*Likelihood ratio test*: χ*^2^*[1] = 0.003; *P =* 0.96). The frequency of agitated movements did not significantly change when visitors where introduced (*B* = 0.00006, *t*[218.3] = 0.103; *P* = 0.92, 95% CI [–0.001, 0.0001]).

### Faecal consistency

Of the faecal samples collected during lockdown, 40.87% were abnormally loose or solid. During the visitor introduction phase these abnormal stools comprised 34.74% of the faecal samples. The decrease in the odds (*Odds ratio =* 0.84, *z* = –0.56; *P =* 0.58, 95% CI [0.44, 1.57]) of abnormal faecal consistency scores during the visitor introduction phase when compared to the lockdown was not statistically significant. Being on-exhibit the day before did not significantly increase the likelihood of seeing abnormal faecal consistency (*Odds ratio* = 0.71, *z* = –0.82; *P =* 0.42, 95% CI [0.31, 1.61]). In other words, there was no evidence from faecal consistency scores that the incidence of abnormal stools was associated with visitor reintroduction (*Likelihood ratio test:* χ^2^[2] = 0.92; *P* = 0.63).

### Faecal cortisol metabolite

We fitted a linear model to explain the variability of dry faecal glucocorticoid metabolites as a function of the interaction between the phases of the study (lockdown vs visitor) and being on-exhibit 24 h prior to the faecal output. The interaction model did not improve the fit of the model when compared with a model without an interaction between exhibit presence and phase (*Likelihood ratio test:* χ^2^[1] = 0.01; *P* = 0.91). There was no significant interaction between the phases of the study and being on-exhibit 24 h prior to the faecal output. The concentration of faecal glucocorticoid metabolites did not significantly increase during visitor reintroduction whether or not the animal was on-exhibit 24 h prior to faecal sample collection *t*[188.27] *=* 0.1; *P* = 0.92. When the orangutans had been on-exhibit 24 h prior to faecal sample collection, the model implied mean FGM concentration during the lockdown phase was 76.42 ng g^–1^ (95% CI [54.52, 98.32]) whereas the mean FGM concentration during the visitor reintroduction phase was 86.57 ng g^–1^ (95% CI [61.28, 111.86]). By contrast, when the orangutans had been in the holding 24 h prior to faecal sample collection, the model implied mean FGM concentration during the lockdown phase was 82.52 ng g^–1^ (95% CI [65.24, 99.80]), whereas the mean FGM concentration during the visitor reintroduction phase was 91.28 ng g^–1^ (95% CI [73.53, 109.04]).

### Other humans: The effect of keeper presence

To understand if keepers affected the orangutans’ physical space use, we tested the relationship between keeper presence and keeper-directed behaviours by the orangutans. The linear model including both the phase and the keeper presence rate explained a more significant amount of variability in keeper-directed behaviour than just individual differences alone (*Likelihood ratio test:* χ^2^[2] = 75.77; *P* < 0.001). There was a linear relationship between the per-minute rate of keeper presence and the per minute rate of keeper-directed behaviour. Regardless of the phase (i.e. lockdown vs visitor reintroduction), each time the keepers increased their frequency of visits, there was a 0.28 increase in rate of keeper-directed behaviour (*t*[239] = 9.26; *P* < 0.001). This effect is also practically significant (95% CI [0.21, 0.33]). To illustrate, if the keepers were present at the public area ten visits more than usual during an observation period, the orangutans increased their keeper-directed behaviours by 2–3 events more within a period of 10 min. The orangutan exhibit is located at the centre of the pavilion and, thus, keepers regularly passed by even without food or enrichment for the animals.

Interestingly, the orangutans did not actively search for the keepers when the rate of keeper presence was low. The relationship between the rates of keeper presence and scanning behaviour was weak. Regardless of the phase, whenever the keepers increased their frequency of visits, the orangutans performed 0.05 more bouts of scanning per minute (*t*[234.7] = 0.77; *P* = 0.44). However, this model does not significantly explain the variability in the rates of scanning (*Likelihood ratio test:* χ^2^[2] = 4.09; *P* = 0.13). These results are consistent with our finding that the orangutans already chose to spend most of their time at the place where the keepers occasionally appeared in order to provide them with enrichment.

## Discussion

To understand the effect of visitor reintroduction on the welfare of Sumatran orangutans, we measured changes in a suite of behavioural and physiological indicators of welfare among the Sumatran orangutans housed at Toronto Zoo before and after the pandemic lockdown measure was lifted. These changes in behaviours have been historically used to measure negative welfare states in orangutans (Birke 2002; Amrein *et al.* 2014; Bloomfield *et al.* 2015). Overall, we did not find any substantial changes among these variables between the phases of the study indicative of a negative welfare state. The indicators we measured can be grouped into three types: space use; behaviour; and faecal measures. The amount of space used by the orangutans did not decrease between the lockdown and the visitor phase. However, the orangutans decreased their hiding behaviour when the visitors were introduced. The orangutans’ general activity level did not differ meaningfully across the phases of the study; neither did indicators such as self-directed behaviours, agonistic behaviours, idiosyncratic object-directed behaviours (e.g. head-slamming), and agitated movements. Lastly, neither the incidence of abnormal faecal consistency (e.g. constipation or diarrhoea) nor concentration of faecal glucocorticoid metabolites changed during visitor reintroduction. However, the rate of keeper presence was associated with both self-directed behaviour and keeper-directed behaviour suggesting that the effect of extraneous variables affect these indicators. Thus, we had no evidence suggesting that the reintroduction of visitors after a prolonged period due to the lockdown resulted in negative welfare states for the orangutans housed at Toronto Zoo.

Our findings add to the growing body of comparative literature regarding visitor effects on the welfare of captive zoo orangutans found in previous studies (Birke 2002; Choo *et al.* 2011; Amrein *et al.* 2014; Bloomfield *et al.* 2015). Visitor effects are quite complex due to the contextual factors brought about by the zoo environment as well as the design of the studies themselves (Rose *et al.* 2020; Pacheco *et al.* 2024). This makes comparative studies essential. Unlike the previous studies that found increases in indicators (e.g. self-directed behaviour, hiding, faecal glucocorticoid concentration) associated with increasing visitor numbers (e.g. Birke 2002; Amrein *et al.* 2014) within the day or a week, we did not find meaningful changes in these indicators when visitors were reintroduced. In fact, we even found a decrease in hiding among the Toronto Zoo orangutans. This could be due to differences in the way visitor presence was observed. In our study, visitor absence was prolonged due to the lockdown which was then followed by a period of high visitor density during visitor reintroduction. Thus, across the phases, sessions with low and high visitor numbers occurred even when there were husbandry events. This is important because, as seen in other species, keeper presence tends to increase animal activity which, in turn, attracts visitors (in felids: Margulis *et al.* 2003; Pacheco *et al.* 2024). Furthermore, we found that the orangutans’ self-directed behaviours were positively associated with keeper presence and that increase in self-directed behaviours associated with visitor reintroduction was negligible in comparison. It is possible that what Birke (2002) and Amrein *et al.* (2014) observed were effects of increased husbandry events which were correlated with visitor presence similar to what we found in our study. Interestingly, in other great apes such as gorillas and chimpanzees (Chelluri *et al.* 2013), keeper activities were found to be related to an increase in some stress-related behaviours such as agonism but a decrease in self-directed behaviours. It is possible that orangutans displace stress or arousal due to husbandry events differently when compared to more social apes like gorillas and chimpanzees. Nonetheless, given the number of variables that may also explain visitor-related animal behaviour in zoos (e.g. keeper presence, weather), future studies should account for contextual variables in zoos for the observed changes in multiple indicators of welfare to clearly distinguish the implications of visitor presence over and above the effect of extraneous variables on the welfare of animals (Goodenough *et al.* 2019; Rose *et al.* 2020; Pacheco *et al.* 2024).

By using multiple indicators of welfare and accounting for contextual variables, our study also provided comparative findings for studies with conflicting interpretations of the same behavioural changes from previous studies (Birke 2002; Bloomfield *et al.* 2015). For example, Bloomfield *et al.* (2015) found that orangutans approached uncovered windows, which they interpreted as a preference for visitors. These findings appear similar to the increase in looking towards visitors reported by Birke (2002), when visitors were instructed to make noise compared with visitors staying silent. Birke (2002) interpreted these changes as an increase in aggression towards noisy visitors. It is possible to accept both interpretations because of different designs of the study. Bloomfield *et al.* (2015) provided their orangutans control through choice by covering windows and allowing the orangutans to choose where they would like to sit, whilst Birke (2002) applied the visitor noise treatment without the orangutans’ control. Thus, the orangutans in the Bloomfield *et al.* (2015) study could have preferred the visitors when they increased their looking behaviour, whereas the orangutans’ attention in Birke’s (2002) study was caught by uncontrollable visitor noise. Given that the orangutans in the Bloomfield *et al.* (2015) study did not perform abnormal behaviours during their study, this seems to support this interpretation. In the case of our study, where the orangutans were not in control of the increase in visitor presence, neither behavioural nor physiological indices of stress changed in relation to visitor presence which is similar to the findings of Bloomfield *et al.* (2015). However, a key difference between our findings is that the Toronto Zoo orangutans stayed away from the windows and remained closer to the keeper entrance. Thus, we did not find evidence supporting the increase in looking or approach behaviour towards visitors which was found in other studies (Birke 2002; Bloomfield *et al.* 2015). Instead, we found that the Toronto Zoo orangutans directed their visual attention to keepers whenever they were around, yet did not scan around when they were not present. This suggests that the Toronto Zoo orangutans did not need to actively search for the keepers. They only needed to pay attention to them when they were in the vicinity of the orangutan exhibit because the orangutans were already at the location where keepers replenish food and enrichment. This supports the alternative explanation to the Bloomfield *et al.* (2015) findings that the orangutans’ preference for particular areas of their exhibit was not affected by visitors but by other resources the orangutans valued. As our findings also differed from Birke’s (2002) results, despite the uncontrollable visitor number increase in our study, this suggests that there are other factors involved beyond the perceived control of the visitor stimulus that may affect orangutan approach or looking behaviour. Using multiple indicators of welfare and accounting for contextual variables will be helpful in making these comparisons for future studies on visitor effects.

We found certain similarities and differences between the behavioural and physiological changes among the orangutans in our study and the other great apes that were studied in the context of the lockdown (Edes *et al.* 2022; Masman *et al.* 2022; Williams *et al.* 2022). None of the primate species (chimpanzees, gorillas, and bonobos) at Twycross Zoo in the UK showed an increase in the concentration of faecal glucocorticoid metabolites (Williams *et al.* 2022), similar to our findings with orangutans. However, we did not find an initial preference for areas away from visitors unlike the gorillas at St Louis Zoo in the US (Edes *et al.* 2022). Instead, the orangutans in our study preferred the back area irrespective of whether or not visitors were present. However, similar to the orangutans in our study, the space preference of their gorillas may be due to contextual variables. In our case, keeper-directed behaviours and scanning behaviours around keeper presence suggest that the orangutans’ space use was strongly influenced by keeper presence. Similarly, we found no changes in behavioural indicators of stress and activity levels, much like the gorillas at Buffalo Zoo in the US and unlike the gorillas, chimpanzees, and bonobos at the Twycross Zoo. It is typical in research on great apes for there to be considerable variability between individuals. However, it appears that there are also institutional level differences. Nonetheless, it is important to note that no studies, including ours, have found that visitors increased stress in the animals. Instead, the behavioural changes had less clear-cut implications for welfare such as that of activity levels (Williams *et al.* 2022). Thus, there was no clear evidence for a negative effect of visitor reintroduction among great apes, in the context of the COVID-19 lockdowns.

### Study limitations

Interpreting the present findings requires several considerations. First, visitor-orangutan relationships may be different in other captive contexts. As mentioned above, there could be differences between study sites that affect the observed changes in behaviour. There are variables beyond keeper factors that vary across institutions, such as where the animals originated. Previous experience of poaching could negatively affect orangutan relationships with humans. Only one of our orangutans, Puppe, came from the wild whilst the rest were born at Toronto Zoo. Interestingly, Puppe also spent the most time at the viewing windows. However, she had spent many decades under human care at Toronto Zoo. Nonetheless, comparing our findings to those of other research centres and sanctuaries will help determine other important contextual variables that could influence visitor effects.

Second, there are still no established scales for behavioural measures of stress. For example, self-directed behaviours have been validated as measures of arousal through pharmacological studies in macaques (Schino *et al.* 1991; Troisi 2002). This has thus become accepted as a measure of frustration, anxiety, stress, or arousal in primates because it appears in contexts such as social conflict or even mental illness in humans (for a review, see Troisi 2002). However, our findings pose the question: how much change warrants concern? Are statistically significant changes actually meaningful to the animals involved? We found that the Toronto Zoo orangutans performed fewer self-directed behaviours than chimpanzees or macaques (Schino *et al.* 1991; Botero *et al.* 2013). Orangutans in an enrichment-focused study (Perdue *et al.* 2012) performed more self-directed behaviours than our participants during visitor introduction. It appears that there is significant variability in the baseline frequencies of self-directed behaviour that may be tied to species, rearing history and primates’ individual experiences. This underscores the need for normative measures of arousal and stress across zoos for each primate species as a benchmark to assess the practical significance of the effects. For the orangutans in our study, given the very low increase (0.01 bouts per min) in the rate of self-directed behaviour in the context of visitor effects, we did not deem the increase to be substantial. Furthermore, we similarly found no association between the phase and the proportion of scans that the orangutans were performing self-directed behaviours suggesting that the orangutans did not increase their bout length of self-directed behaviour when visitors were present. Therefore, despite the statistically significant increase in the rate of self-directed behaviour, this effect was not substantial and we did not deem it practically meaningful.

Finally, causality cannot be inferred from our findings. The lockdown isolated the effects of visitors from the effects of time and routine. However, the lockdowns were also unpredictable and did not allow for reversal testing. Hence, the order of the phases could have been a confounding variable in this study. Visitor numbers fluctuate across the year at Toronto Zoo. An alternative interpretation of our findings could be that the orangutans were already stressed during the lockdown in anticipation of visitors (i.e. a ceiling effect). Both phases of the study occurred in the peak visitor season (May–August). A reversal phase would have allowed us to test if the indicators of stress remained the same in non-peak season. Nonetheless, the average levels of indicators across phases were close to zero. Thus, it is unlikely that the lack of changes in indicators of stress were due to a ceiling effect.

### Animal welfare implications

This study has two important implications for the welfare of orangutans in zoos. First, we did not find supporting evidence for the negative effects of visitor reintroduction among the orangutans at Toronto Zoo. However, proving the null hypothesis that visitors have no effect on the welfare of orangutans is impossible and is beyond the limits of what we can conclude from our study. There *can* still be effects with respect to other stimuli associated with visitor presence (e.g. noise [Birke 2002], food provision [Choo *et al.* 2011]) and these warrant further investigation. It is important for future visitor effect studies to disentangle the effects of stimuli that visitors can cause (e.g. noise) and stimuli simply associated with visitors (e.g. husbandry events, keeper talks) to ensure appropriate interventions in zoos. Many of the concerns regarding zoo visitors rest on balancing the negative effects of visitors on zoo animal welfare with conservation education (Hutchins *et al.* 2003; Whitworth 2012; Carr 2016; MacDonald & Ritvo 2016b; Patrick & Caplow 2018). If the negative effects of visitors are not inextricably linked to their presence, then behavioural adjustments of visitors can allow conservation education to continue whilst eliminating any negative welfare effects of such programmes.

Second, our findings showed that for the orangutans in our study, the effects of the keepers’ presence seem to matter to their behaviour. The effects of keepers on orangutan welfare should receive more research and management focus given the keepers’ consistent daily presence throughout the lives of animals under human care. Orangutans under human care approach novel items more frequently than orangutans in the wild. Forss *et al.* (2015) argued that orangutans under human care are often given novel items that do not have negative consequences. They further asserted that because of this positive interaction, the orangutans tend to trust their keepers. In the case of the orangutans at Toronto Zoo, the evidence suggests that the keepers also affect their behaviours. The orangutans preferred to stay where the keepers appeared for supplementation of enrichment and also increased self-directed behaviours in response to increasing keeper presence. It is possible that both the resources brought by the keepers and interaction between the keepers and the orangutans reinforced this spatial preference. Yet, despite the spatial preference of the orangutans, the increase in self-directed behaviour could still signal a potential welfare concern. Self-directed behaviours typically appear in situations where there is motivational conflict or a goal-directed behaviour has been thwarted (Troisi 2002). Thus, this could represent a limit of control that the orangutans at Toronto Zoo have over food, enrichment, and keeper interaction. This implies that the orangutans could benefit from a more stimulating and dynamic environment that provides challenges to the orangutans, whilst simultaneously offering them opportunities to exercise agency. This of course is not unique to Toronto Zoo, especially in the case of captive great apes that require plenty of cognitive stimulation due to their advanced cognitive abilities (Clark 2011). Strategic management of both food resources, keeper interaction, and cognitive stimulation could potentially help encourage the zoo orangutans to use more of their space and focus less on the keepers.

## Conclusion

Overall, we found no evidence suggesting that the welfare of the six Sumatran orangutans in our study was negatively affected by the reintroduction of visitors after prolonged absence due to the lockdown. No substantial changes were found among either behavioural or physiological indicators of stress indicating negative welfare effects. The orangutans also did not change their use of space. While visitors did not seem to be important to the orangutans’ daily lives, we did find that keeper presence affected the orangutans’ behaviour. Thus, future study is warranted on how keeper variables affect the welfare of Sumatran orangutans in zoos.
